# Prognostic significance of combined Lymphocyte-monocyte Ratio and Tumor-associated Macrophages in Gastric Cancer Patients after Radical Resection

**DOI:** 10.7150/jca.44440

**Published:** 2020-06-28

**Authors:** Bin-bin Xu, Yu Xu, Jun Lu, Yuan Wu, Jia-bin Wang, Jian-xian Lin, Jian-wei Xie, Ping Li, Chao-hui Zheng, Ai-min Huang, Chang-ming Huang

**Affiliations:** 1 Department of Gastric Surgery, Fujian Medical University Union Hospital, Fuzhou, China.; 2 Department of General Surgery, Fujian Medical University Union Hospital, Fuzhou, China.; 3 Key Laboratory of Ministry of Education of Gastrointestinal Cancer, Fujian Medical University, Fuzhou, China.; 4 Department of Pathology, the School of Basic Medical Sciences, Fujian Medical University.

**Keywords:** gastric cancer, lymphocyte-monocyte ratio, tumor-associated macrophage, recurrence, prognosis

## Abstract

**Background:** Immune function is recognized as an important prognostic indicator in gastric cancer (GC). The relationship between the lymphocyte-monocyte ratio (LMR) and tumor-associated macrophage (TAM) has received far less attention.

**Methods:** A total of 401 patients from a prospective trial (NCT02327481) were enrolled in this study. The relationships between the LMR, TAM, and clinicopathologic variables were analyzed using a Kaplan-Meier log-rank survival analysis, and multivariate Cox regression models were used to identify associations with recurrence-free survival (RFS) and overall survival (OS). The discriminatory power of the prognostic models for both RFS and OS were compared. The decision curve analysis was performed to compare the clinical utility of the prognostic models.

**Results:** High LMR was observed in 81.5% of the 401 GC patients, and high TAM infiltration was observed in 45.9% of the patients. In a multivariate Cox analysis of all patients, LMR and TAM were both independent prognostic factors for RFS and OS. Patients with high TAM expression had similar mean LMR levels than patients with low TAM expression. Moreover, LMR appeared to lose its prognostic significance in patients with high TAM expression levels. Finally, the model that included the TAM had better predictive capability and clinical utility for both RFS and OS.

**Conclusions:** Although LMR and TAM are both independent predictors of RFS and OS in resectable GC patients, LMR seem to attenuate its prognostic significance in patients with high TAM expression. This information may be helpful in the clinical management of patients with GC. Further external studies are warranted to confirm this hypothesis.

## Introduction

Gastric cancer (GC) is a major world health burden and is rated as the third leading cause of cancer death and the fifth-most-common cancer worldwide 1. The functional status of the immune system significantly affects the prognosis of many human malignancies, including GC 2. Therefore, we hypothesized that immune biomarkers may enable better stratification of GC patients who could benefit from surgery.

Accordingly, the predictive values of several blood inflammation-immune biomarkers, including systemic immune-inflammatory index (SII), C-reactive protein (CRP), CRP-to-albumin ratio (CAR), prognostic nutrition index (PNI), neutrophil-lymphocyte ratio (NLR), platelet-lymphocyte ratio (PLR), and lymphocyte-monocyte ratio (LMR), have been widely reported for various malignancies 3-5. Recently, Chan et al. reported that the LMR is an independent prognostic marker in patients with resectable colorectal cancer and appears to be superior to the pre-existing biomarkers such as NLR, PLR, and modified Glasgow prognostic score (mGPS) 6. Shen et al. found that LMR had the highest area under the curve (AUC: 0.814) for the prognosis of hepatocellular carcinoma 7. Our previous study showed that combining the LMR and hemoglobin level, a new prognostic score could improve the prediction of clinical outcomes for patients with GC 8.

Tumor-associated macrophages (TAMs), which are derived from circulating monocyte populations, are one of the most important inflammatory immune cells in the tumor microenvironment; they encourage metastasis and tumor progression and are usually marked by CD68 9, 10. Zhou et al. found that TAMs have an important role in maintaining the inflammation process during gastric carcinogenesis 11. However, the relationship between LMR and TAMs has not been well investigated in GC despite evidence implicating both in monocyte levels and tumor carcinogenesis.

In light of these recent findings, the present study aimed to investigate the prognostic value of LMR and TAMs in GC. Although a few publications have demonstrated that the combination of LMR and TAMs can be a useful prognostic factor for Hodgkin lymphoma 12, 13, to the best of our knowledge, this is the first study that aimed to determine whether TAMs status influences the prognostic role of LMR in cancer patients.

## Materials and Methods

### Patients

A cohort of 419 patients was included in the final analysis from a total of 438 patients who enrolled in a prospective trial at a high volume tertiary referral center in China (Fujian Medical University Union Hospital). The trial was registered at ClinicalTrials.gov (NCT02327481). The details regarding inclusion, exclusion, quality control and randomization have been previously reported 14. The present study is a substudy of the above clinical trial. After excluding 10 patients with neuroendocrine carcinoma, 6 patients who underwent palliative surgery and 2 patients without evidence of GC, the present analysis was restricted to 401 patients for whom curative gastrectomies were performed and for whom the postoperative pathology confirmed stage I, II, or III gastric adenocarcinoma (pT1-4aN0-3M0) according to the 7th American Joint Committee on Cancer staging 15. All patients had undergone R0 laparoscopic gastrectomy between January 1, 2015 and April 1, 2016. Fluoride-based adjuvant chemotherapy is routinely recommended for most stage II or III GC patients, but depending on the patient's wishes and physical condition 16. Postoperative adjuvant chemotherapy (PAC) was categorized as received or not received 17, 18. Postoperative follow-up was performed every 3 months for 2 years and then every 6 months between years 3 and 5. Most routine patient follow-up appointments included a physical examination, laboratory tests, chest radiography, abdominal ultrasonography or CT and an annual endoscopic examination.

### Blood inflammatory measures

Patients routinely received blood tests throughout the 7 days before surgery. LMR was defined as the ratio of the absolute blood lymphocyte count to the absolute blood count of monocytes. The optimal cutoff value was identified as 3.15 for blood LMR, according to our previous study 19. Patients were then dichotomized into “low” and “high” groups, in which “low” was less than or equal to the cutoff value and “high” was greater than the cutoff value.

### Immunohistochemistry

The contents of infiltrated macrophages, in the individual GC specimens were characterized by IHC using an avidin-biotin peroxidase complex method, as previously reported 20. Briefly, slides (4 μm thick consecutive paraffin sections) from the blocks with the highest tumor content for each sample were used for immunohistochemical staining and immersed in xylene and rehydrated through graded concentrations of ethanol followed by PBS buffer and deionized water for 5 min each. Slides were then heated to 100 °C for 20 min in a pH 9 Tris-based solution. All slides were incubated with the primary antibodies for 60 min at 37 °C for 1 h (dilutions: mouse anti-CD68 1:500, MAIXIB.BIO, China) and were then washed. A secondary antibody for mouse IgG was added for 30 min and the slides were again washed. The sections were processed with the universal SP Elivision-plus kit (MAIXIB.BIO, Fuzhou, China). Next, the sections were counterstained with hematoxylin.

All individual GC specimens were evaluated by two pathologists (Y. X and AM. H), who were blinded to the patient characteristics and outcomes. Using highpower microscopy, the semi-quantitative immunohistochemical grading of TAMs in tumors was determined. In brief, we selected five fields with the richest infiltration of GC calculated the mean percentages of TAMs. First, a quantitative score on the basis of the estimated percentage of immunopositive-stained cells among total cells was specified based on the following rule: 1 (< 1% cells); 2 (1-10% cells); 3 (11-33% cells); 4 (34-66% cells); and 5 (67-100% cells). We defined the Immunopositive cells as those showing partial or complete staining within the cytoplasm and/or plasma membrane. Then, staining intensity was recorded and scored as follows: 0 (none), 1+ (mild), 2+ (moderate), and 3+ (intense). Finally, scores (ranging from 1 to 8) were calculated by adding the percentage positivity scores and the intensity scores for each section. Accordingly, the patient cohort was divided into two groups with high or low CD68+ expression, which was previously reported 16 (Figure S1).

### Statistical Analysis

The significance of the differences between the categorical variables were analyzed by χ^2^ tests. We considered recurrence-free survival (RFS) as the time from the date of surgery until recurrence or the last follow up and overall survival (OS) as the time from the date from surgery to the date of death due to any cause or the date of the last follow-up. Differences in RFS and OS were evaluated by the log-rank test and were described by the Kaplan-Meier method. To identify significant predictors, multivariate survival analysis and the calculation of hazard ratios (HRs) were performed using a Cox proportional hazards model, in which all significant variables in the univariate model were used. The Akaike information criterion (AIC) could asymptotically select the model which minimizes mean squared error of prediction or estimation 21, 22. Bayesian information criterion (BIC) is a simple expression which involves the maximized likelihood, the sample size, and the number of risk factors in the model. It could be used to approximate the posterior model probabilities 23, 24. The Harrell index of concordance (C-index) was used to evaluate the discriminative ability of the prognostic models 25. In the present study, we used the AIC, BIC and C-index to compare the accuracy of the different prognostic models. The decision curve analysis was performed to compare the clinical utility of the prognostic models 26. All tests were two-sided, and p < 0.05 was considered statistically significant. Statistical analysis was performed using SPSS version 19 (SPSS Inc., Chicago, IL) and R version 3.1.2 (R Foundation for Statistical Computing, Vienna, Austria). The R package “CsChange” was used to calculate the difference of C-indices between two different models 25.

## Results

### Clinicopathological Characteristics of the Patients

The patients' baseline parameters and the correlations between LMR, TAM and the clinicopathological characteristics of the patients are shown in Table 1. There was no significant difference between the low and high LMR or TAM groups in gender, age, differentiation, tumor depth, lymph node metastasis or TNM stage.

### Survival Data

**Figure 1:**
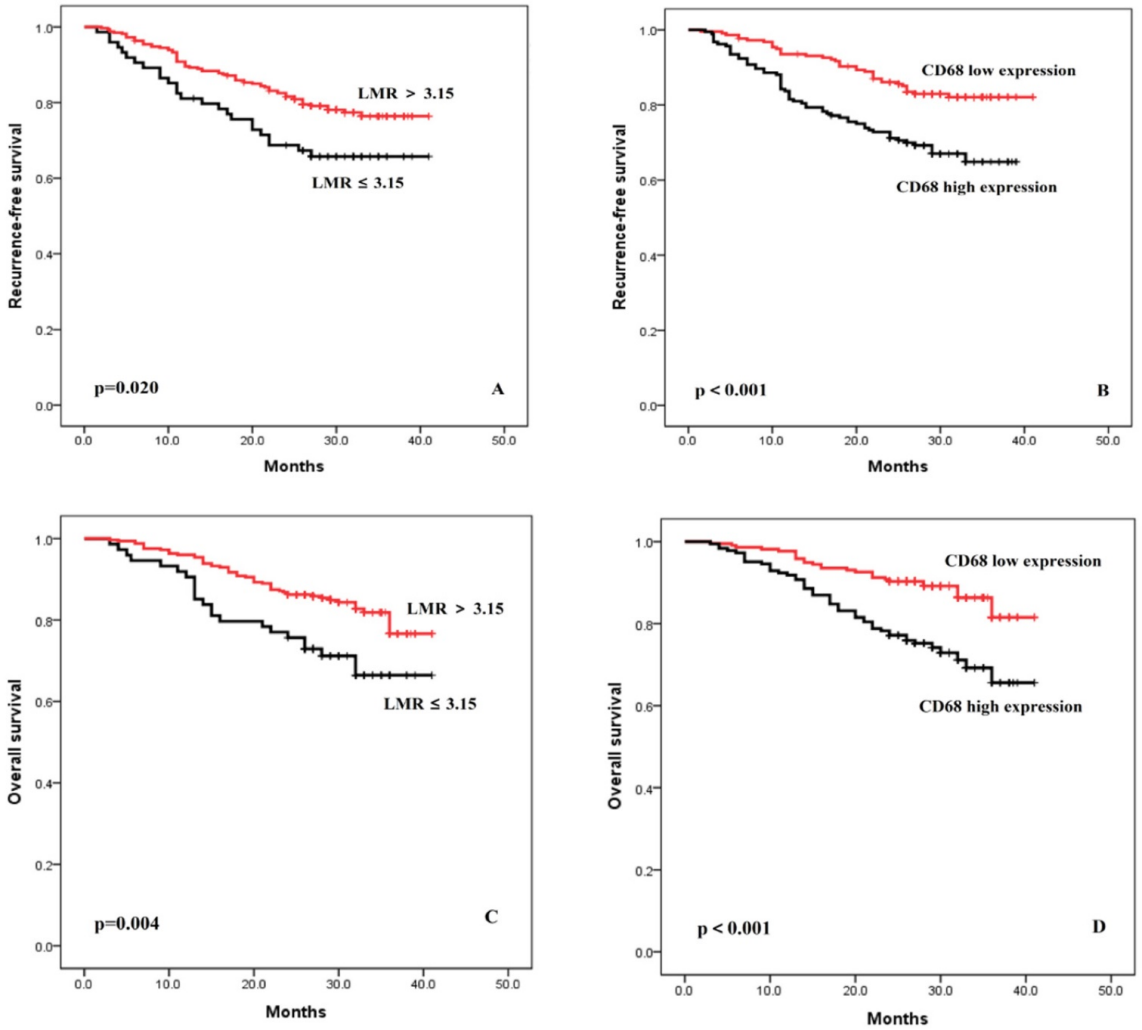
Recurrence-free survival and overall survival curves after surgery, stratified according to LMR (**A and C**) and TAM (**B and D**).

The median follow-up period was 29 months (range 3-41 months). Patients with lower LMR had poorer prognoses. As shown in Figure 1A, the 3-year RFS of the group with a LMR ≤3.15 and >3.15 were 65.7% and 77.4%, respectively (p = 0.020). Patients with a higher expression of TAM had poorer prognoses. As shown in Figure 1B, the 3-year RFS of the group with high expression of TAM and low expression of TAM were 64.9% and 82.1%, respectively (p < 0.001). Similar trends were found in the OS analysis (Figure 1C-D).

### Independent Predictors of Survival

A univariate analyses of RFS revealed that tumor location (p = 0.044), tumor size (p < 0.001), tumor differentiation (p = 0.001), lymphovascular involvement (p < 0.001), TNM stage (p < 0.001), PAC (p = 0.001), LMR (p = 0.020) and TAM (p < 0.001) were significantly associated with RFS. Subsequent multivariate analyses revealed that lymphovascular involvement, TNM stage, LMR and TAM were independent prognostic factors for RFS, while tumor location, tumor size, tumor differentiation, and PAC were not (Table 2). Similar results were found in the univariate and multivariate analyses of OS (Table 2).

### Effects of TAM Levels on the LMR

**Figure 2:**
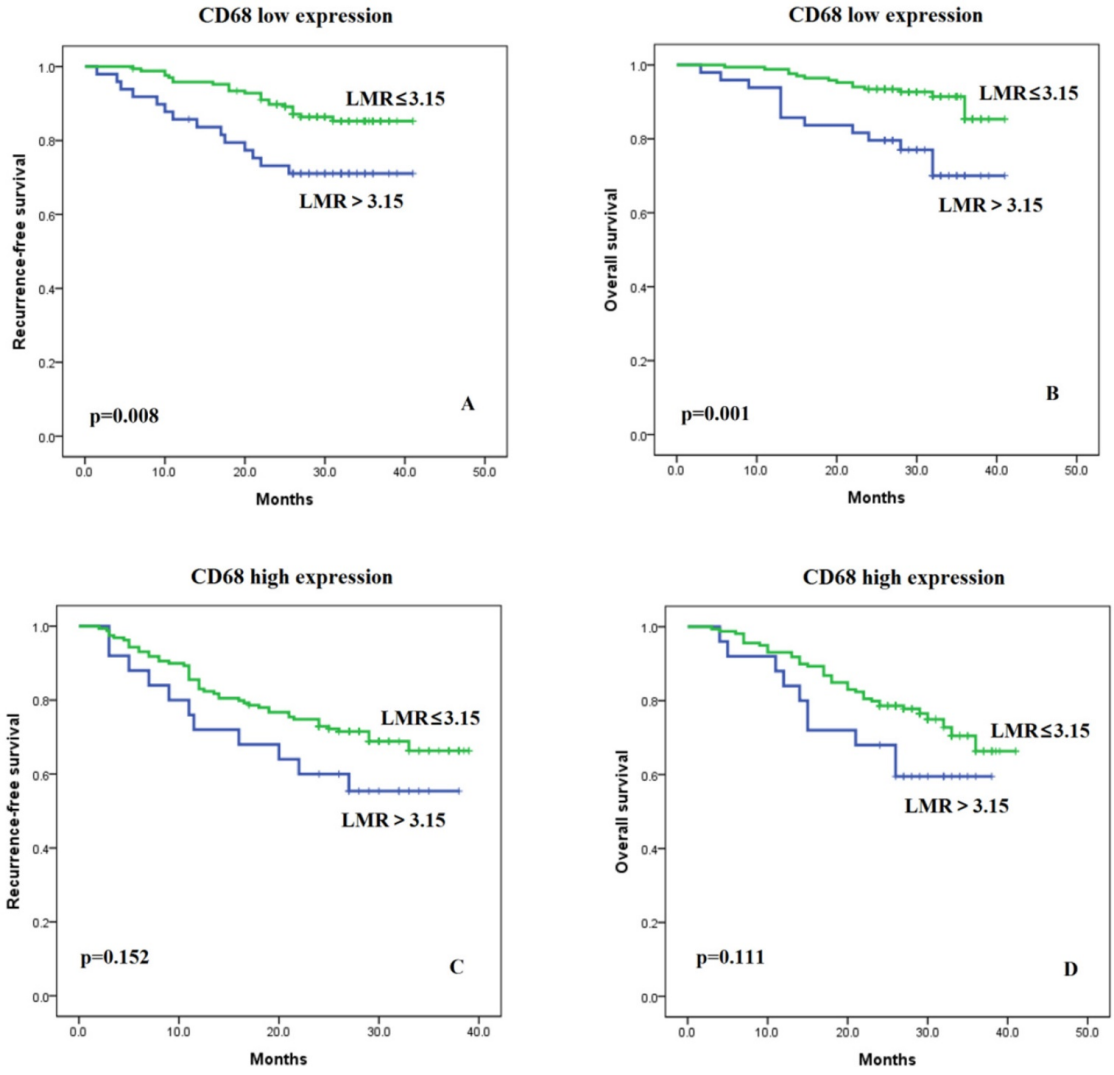
Kaplan-Meier curves of recurrence-free and overall survival in patients with low LMR (≤3.15) versus high LMR (>3.15) in cases of low (**A and B**) and high TAM (**C and D**) levels.

There were no significant differences in LMR level between the high and low TAM infiltration cohorts (LMR median: 4.6 vs. 4.7, p=0.615) (Figure S2). The Pearson Correlation Coefficient for the two variables was -0.018, p=0.720. Furthermore, we examined whether the association of LMR with RFS and OS depended on TAM status. We found that the LMR was more strongly associated with RFS and OS in patients with low TAM levels (Figure 2A and 2B, p=0.008 and p=0.001, respectively). Conversely, the association of LMR with RFS and OS was weaker in the high TAM expression group (Figure 2C and 2D, p=0.152 and p=0.111, respectively). In addition, we divided the patients into four different groups: LMR high and TAM low, LMR low and TAM low, LMR high and TAM high, LMR low and TAM high (Figure S3A). And we found that patients with “LMR high and TAM low” group had the best prognosis when compared with the other three groups for RFS (all p<0.05). Patients in the other three groups had similar prognosis (all p>0.05), although patients in the “LMR low and TAM high” appeared to have the worst prognosis. The same findings were observed in the analyses for OS (Figure S3B).

### Comparison of the Predictive Capability of the 2 Models

Two prognostic models, one with and one without the TAM were created. The two models were compared using the C-index, AIC, and BIC. A higher C-index value and a lower AIC or BIC value indicated a better predictive capability. The model with the higher C-index and the lower AIC or BIC was the model with the TAM included for both the RFS and OS analyses (Table 3). In addition, we calculated the C-indices for the four models, TNM, TNM+LMR, TNM+TAM and TNM+LMR+TAM, and showed the results in Table S1. When compared with the TNM, we found that the C-indices for the other three models increased gradually, and all the differences were significantly. As expected, the model with highest predictive value is TNM+LMR+TAM.

### Comparison of the Clinical Utility of the 2 Models

**Figure 3:**
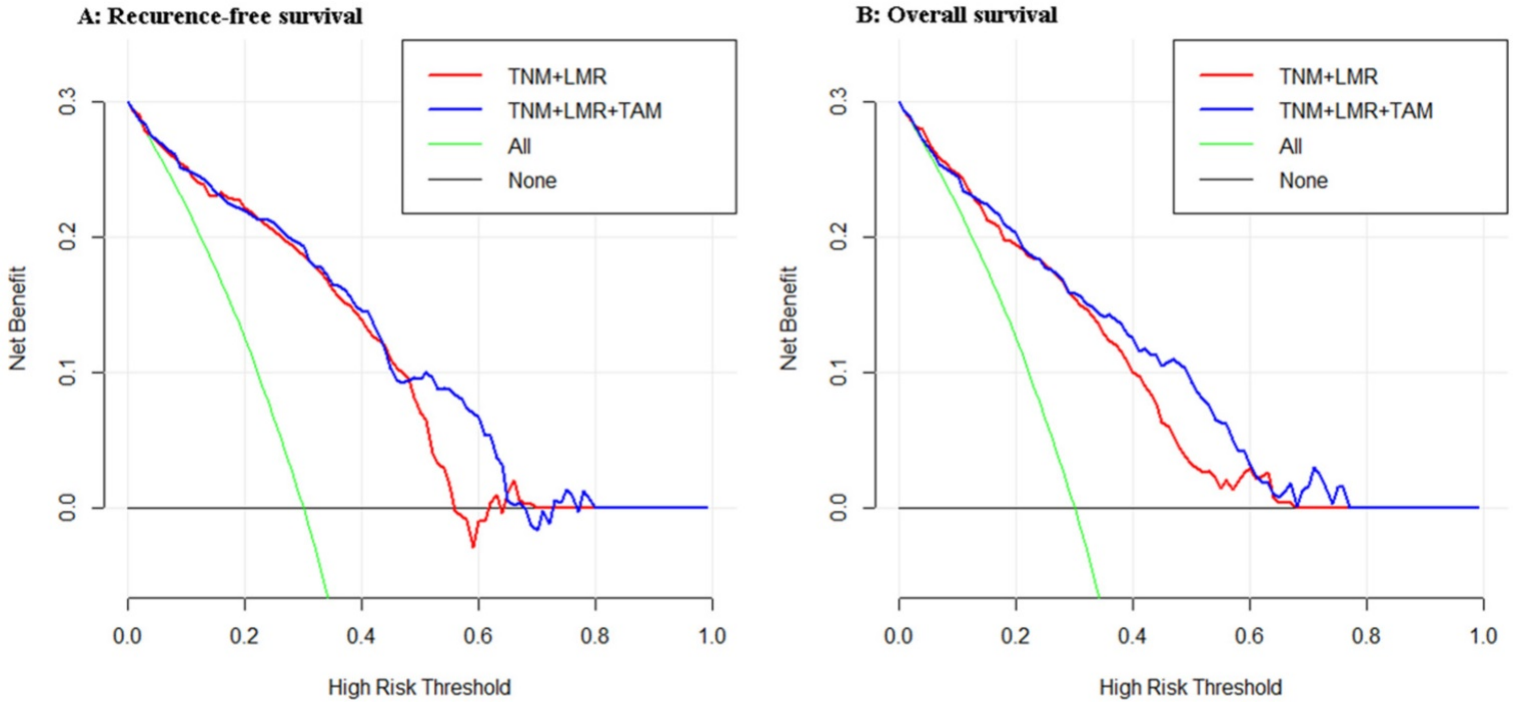
Decision curve analyses for the two models for (**A**) recurrence-free survival and (**B**) overall survival in patients with gastric cancer after radical gastrectomy. The x-axis means the risk threshold probability which changes from 0 to 1. The y-axis shows the calculated net benefit corresponding to a given threshold probability. The blue curve and red curve depict the net benefit of the two model-based selection strategies for screening. The green and black lines display the net benefits in the alternative strategies of screening all patients (green) versus screening no patients (black) in the data set.

As shown in Figure 3, we compared the net benefit between the two models (TNM+LMR and TNM+LMR+TAM). It implies that if we use a risk threshold probability (e.g. 55%), so that screening is recommended if an individual's risk is above the given threshold. As for the calculated net benefit (the weighted sum of true positives subtracted by the number of false positives), it is larger for the prediction model with TAM than it is in the strategies that use the model without TAM or do not use any models (None), for both the RFS and OS analyses.

## Discussion

The present study described that lymphocyte-to-monocyte ratio (LMR) and tumor-associated macrophages (TAMs) were strongly associated with poor RFS and OS after R0 gastrectomy for gastric cancer (GC). Importantly, we found that the LMR may lose its prognostic value in the setting of high CD68+ TAM expression. In addition, the inclusion of TAM substantially improved the prognostic value of the TNM-LMR-based model. This is the first report about the influence of TAM status on the prognostic value of LMR.

CD68 has been widely used as a specific marker of TAMs in tumor tissues 27, 28. Several studies have reported the clinical and functional significance of CD68+ TAMs in gastric cancer, though without differentiating the M1 and M2 subsets 29, 30. Therefore, we did not address the M1 or M2 subsets in the present study either. We added the above description in the Discussion section with red mark.

Monocytes play a prominent role in human malignancy-related immunology. Monocytes can promote tumorigenesis and angiogenesis and can also inhibit the antitumor immune response *in vivo* 31. Based on this knowledge, the lymphocyte-monocyte ratio, a simple derivative of routine blood counts, was further identified as an important prognostic indicator in patients with various solid tumors. Stotz et al. 32 reported that LMR was an independent prognostic marker of time to recurrence and overall survival in stage III colon cancer. Goto et al. reported 33 that LMR was significantly associated with disease-free survival in breast cancer patients who received neoadjuvant chemotherapy. Chan et al. 6 found that the LMR was better than pre-existing biomarkers such as NLR, PLR, and mGPS. However, the optimal cutoff point of these markers, including LMR in patients with different stages of solid tumors, remains poorly defined 4, 6.

Chan et al. 6 indicated that the optimal cutoff point for LMR varies among stage I to III colorectal cancer; specifically, the optimal cutoff points in stage I, II, and III are 2.13, 1.59, and 2.38, respectively. To date, only a few studies have explored the prognostic role of the preoperative LMR in patients with different stages of GC, and different cutoff values of LMR were applied in these studies 19, 34, 35. Hsu et al. collected data of patients with GC between 2005 and 2010 and defined the cutoff value as 4.8 34. Zhou et al. used 4.32 as the cutoff value for stage II/III gastric cancer 35. Compared with the above study, our previous study included more patients (n=1800) from 2008 to 2013. In addition, the cutoff value was internal validated 19. Therefore, in this study, we used the cutoff value that we had previously reported 19, and confirmed that LMR, with 3.15 as the cutoff value was an independent predictor of RFS and OS in resectable GC. In addition, our previous study using bulk data found that LMR had the highest predictive value when compared with the absolute monocyte count, NLR, PLR and other biomarkers. To further improve the predictive value of clinical outcomes, the authors combined the LMR and the hemoglobin and created a novel prognostic marker named complete blood count-based inflammatory score (CBCS), which showed a better predictive value 8. However, in the present study, we focused on exploring the relationship between LMR and TAM in GC.

Moreover, serum monocytes could be recruited into the microenvironment and differentiate into TAMs 36, 37. One of the main theoretical bases for the establishment of the LMR is that an elevated circulating monocyte level may reflect an increased production of TAMs, which can act as a marker of high tumor burden. In other words, circulating monocytes need to be recruited in tumor tissues first and differentiate into tumor-associated macrophages (TAMs), exerting their pro-tumoral action thereafter 4. Therefore, TAM was directly measured in this study to more accurately determine the inflammatory immune response in the microenvironment of patients with tumors.

Studies have shown that cancer patients with high infiltration of TAMs have a poor prognosis 10, 38, 39. In GC patients, TAMs have shown great potential in the formation of more individual prognoses and thus have great potential as biomarkers for evaluating GC staging and progression 40. TAMs may lead to worse outcomes through the following potential mechanisms: First, TAM itself promotes tumor angiogenesis 41. Second, TAM-derived cytokines, such as IL-6, which may enter circulation and influence the systemic inflammation status, significantly contribute to inflammation, proliferation, immunosuppression, and angiogenesis 42, 43.

Interestingly, a prior study found that the local immune tumor microenvironment (TME) and systemic inflammation were related 33. Koh et al. indicated that LMR and TAM are two parameters that reflect the systemic immunity of the host and the tumor microenvironment 12. However, previous studies did not investigate the influence of LMR on TAM 3, 12, 19. Therefore, further evaluation of TME markers such as TAMs, evaluation of systemic inflammatory markers such as LMR, more accurate identification of patient-specific immune status and prediction of prognosis is necessary 33.

Based on the above evidence, the second part of the study was conducted to define the prognostic ability of LMR in different GC patients according to TAM status. We were surprised to find that LMR had different prognostic abilities in different groups classified by TAM, although LMR level was not associated with TAM status. In our study, we show that the LMR has the potential to be a prognostic factor but only when TAM expression is low. Compared with TNM+LMR, the prognostic model based on TAM and TNM+LMR demonstrated significantly better discriminatory ability and clinical utility for both RFS and OS. These findings may have implications for clinical practice. First, our results indicate that the well-established LMR should be used with caution in cases of high TAM infiltration, at least until further prospective studies confirm or reject this hypothesis. In addition, understanding the TME of primary GC can guide future trials. Although the mechanism underlying these findings is complex and remains unclear, our results here may be due to, at least in part, the very important immunomodulatory effects that TAM possesses in tumor progression.

In the present study, we focused on the prognostic value of LMR and TAMs in GC and whether TAMs status influences the prognostic role of LMR in cancer patients. The ratio of LMR based on the IHC staining staining in tumor tissues were not available in this study. The hypothesis that the association between the LMR in blood and in tumor tissues is interesting. In addition, as we know, no study reported the association between the ratio of LMR in tumor tissues and in blood. Further study is needed to explore the association.

Our study had several limitations. First, this study was conducted at a single hospital and was retrospective in nature with small sample size. Therefore, whether the cutoff values of LMR and TAM could be used for all GC patient needs to be validated in larger, multicenter studies. Second, various biomarkers were confirmed to be related to the prognosis for patients with GC. In the present study, we only chose LMR as the biomarker and investigated the relationship between LMR and TAMs in GC. Third, the prognostic value of blood monocyte count was not evaluated; also, as an unresolved issue nowadays 10, 44, 45, these specimens as well as semi-quantitative IHC evaluations may still not completely reflecting the tumor immune microenvironmental status. Fourth, the present study had a relatively short follow-up period. However, when it occurs, recurrence of GC usually develops within the first 2 years after surgery. Finally, although we did not explore the mechanism underlying these findings, the present study may provide the base for further research. In addition, this is the first study to examine the interaction between LMR and TAM in patients with resectable GC.

## Conclusions

To the best of our knowledge, the present study is the first to explore the interaction between prognostic values of LMR and TAM by using prospectively acquired data from a clinical trial in which surgical management and pathological assessment adhered to strict quality measures. We first found that the LMR may lose its prognostic value in cases of high TAM expression. Thus, we should pay close attention to LMR and TAM, and we expect that low-cost markers will facilitate personalized multidisciplinary treatments and surveillance for GC patients. Considering the limitations, further studies that obtain data from a prospective multi-institute study with a large number of patients and basic research in cell and animal models are needed to confirm these conclusions.

## Supplementary Material

Supplementary figures and tables.

## Figures and Tables

**Table 1:** Patients Characteristics According to the LMR and TAM

Characteristics	LMR			TAM		
	Low (%)	High (%)	p value	Low expression (%)	High expression (%)	*p* value
All cases	74(18.5)	327(81.5)		217(54.1)	184(45.9)	
**Sex**			0.492			0.749
Female	21(28.4)	109(33.3)		72(33.2)	58(31.5)	
Male	53(71.6)	218(66.7)		145(66.8)	126(68.5)	
**Age (y)**			0.167			0.668
<65	45(60.8)	228(69.7)		150(69.1)	123(66.8)	
≥65	29(39.2)	99(30.3)		67(30.9)	61(33.2)	
**Tumor location**			0.443			0.330
Proximal	21(28.4)	96(29.4)		68(31.3)	49(26.6)	
Middle	16(21.6)	53(16.2)		37(17.1)	32(17.4)	
Distal	35(47.3)	156(47.7)		103(47.5)	88(47.8)	
Entire	2(2.7)	22(6.7)		9(4.1)	15(8.2)	
**Tumor size (cm)**			0.114			0.441
<5	39(52.7)	206(63.0)		137(63.1)	108(58.7)	
≥5	35(47.3)	121(37.0)		80(36.9)	76(41.3)	
**Differentiation**			0.120			0.840
Differentiated	37(50.0)	131(40.1)		92(42.4)	76(41.3)	
Undifferentiated	37(50.0)	196(59.9)		125(57.6)	108(58.7)	
**Lymphovascular invasion**			0.897			0.130
Absent	43(58.1)	185(56.6)		131(60.4)	97(52.7)	
Present	31(41.9)	142(43.4)		86(39.6)	87(47.3)	
**Tumor depth**			0.614			0.225
T1/2	28(37.9)	140(42.8)		97(44.7)	71(38.6)	
T3/4	46(62.1)	187(57.2)		120(55.3)	113(61.4)	
**Lymph node metastasis**			0.238			0.919
Absent	24(32.4)	134(41.0)		86(39.6)	72(39.1)	
Present	50(67.6)	193(59.0)		131(60.4)	112(60.9)	
**Pathological stage**			0.476			0.561
I	21(28.4)	114(34.9)		78(35.9)	57(31.0)	
II	15(20.3)	69(21.1)		43(19.8)	41(22.3)	
III	38(51.4)	144(44.0)		96(44.2)	86(46.7)	
**Adjuvant chemotherapy**			0.593			0.917
No	29(39.2)	116(35.5)		79(36.4)	66(35.9)	
Yes	45(60.8)	211(64.5)		138(63.6)	118(64.1)	

**Table 2:** Prognostic Factors for Survival Identified by Univariate and Multivariate Analyses

RFS	Univariate			Multivariable		
Variables	Hazard Ratio	95% CI	*p* value	Hazard Ratio	95% CI	*p* value
Sex (male)	1.246	0.779-1.945	0.332			
Age (≥65)	1.182	0.776-1.801	0.436			
Tumor location (lower third)	0.807	0.655-0.994	0.044	1.057	0.870-1.284	0.577
Tumor size (≥5cm)	3.792	2.480-5.800	< 0.001	1.288	0.824-2.013	0.268
Tumor differentiation (undifferentiated)	2.130	1.357-3.341	0.001	1.111	0.688-1.795	0.666
Lymphovascular involvement	6.354	3.877-10.416	< 0.001	2.065	1.220-3.493	0.007
Pathological stage (stage III)	6.547	3.910-10.964	< 0.001	5.278	2.985-9.332	< 0.001
Postoperative Adjuvant chemotherapy (No)	2.298	1.404-3.761	0.001	1.513	0.900-2.546	0.188
Low LMR (≤3.15)	1.707	1.082-2.693	0.020	1.929	1.171-3.177	0.010
High TAM infiltration	2.150	1.424-3.245	< 0.001	2.451	1.589-3.780	< 0.001
**OS**						
	**Univariate**			**Multivariable**		
**Variables**	**Hazard Ratio**	**95% CI**	***p* value**	**Hazard Ratio**	**95% CI**	***p* value**
Sex (male)	1.053	0.065-1.704	0.832			
Age (≥65)	1.396	0.884-2.203	0.152			
Tumor location (lower third)	0.924	0.738-1.156	0.488			
Tumor size (≥5cm)	3.581	2.236-5.736	< 0.001	1.410	0.852-2.334	0.181
Tumor differentiation (undifferentiated)	2.115	1.282-3.489	0.003	1.336	0.783-2.280	0.288
Lymphovascular involvement	4.438	2.774-7.100	< 0.001	2.415	1.293-4.510	0.006
Pathological stage (stage III)	6.368	3.624-11.189	< 0.001	3.148	1.807-5.485	< 0.001
Adjuvant chemotherapy (No)	1.929	1.139-3.268	0.014	1.498	0.862-2.603	0.152
Low LMR (≤3.15)	1.993	1.224-3.243	0.006	2.475	1.450-4.226	0.001
High TAM infiltration	2.463	1.548-3.918	< 0.001	2.934	1.803-4.773	< 0.001

**Table 3:** Comparison of the Prognostic Accuracies of Different Models

RFS	Model		*p* value
	TNM+LMR	TNM+(LMR+TAM)	
C-index (95% CI)	0.7994(0.7622-0.8364)	0.8328(0.7988-0.8668)	**0.021**
AIC	936.5622	920.4846	/
BIC	941.5683	928.0341	/
**OS**			
C-index (95% CI)	0.7693(0.7263-0.8123)	0.8036(0.7612-0.8459)	**0.024**
AIC	971.2207	953.2399	/
BIC	978.8185	963.3703	/

C-index indicates Harrell concordance index; AIC indicates Akaike Information Criterion; BIC indicates Bayesian Information Criterion.
