# Supplementary material for: Prognostic significance of combined Lymphocyte-monocyte Ratio and Tumor-associated Macrophages in Gastric Cancer Patients after Radical Resection
Source: J Cancer. 2020 Jun 28;11(17):5078–87. doi: 10.7150/jca.44440 (PMC7378932; doi:10.7150/jca.44440)
Supplement: Supplementary file 1 — Supplementary figures and tables. [file jcav11p5078s1.pdf]

Table S1. Comparison of the Prognostic Accuracies of Different Models

**RFS**	**C-index**	**95% CI**	***p* value***
TNM	0.7428	0.7029-0.7828	/
TNM+LMR	0.7693	0.7263-0.8123	**0.012**
TNM+TAM	0.7917	0.7464-0.8369	**<0.001**
TNM+LMR+TAM	0.8036	0.7612-0.8459	**<0.001**
**OS**	**C-index**	**95% CI**	***p* value***
TNM	0.7699	0.7374-0.8024	/
TNM+LMR	0.7994	0.7622-0.8364	**0.015**
TNM+TAM	0.8127	0.7776-0.8479	**<0.001**
TNM+LMR+TAM	0.8328	0.7988-0.8668	**<0.001**

*Comparison of C-index between the TNM and other prognostic models.

C-index indicates Harrell concordance index

Low CD68+ expression

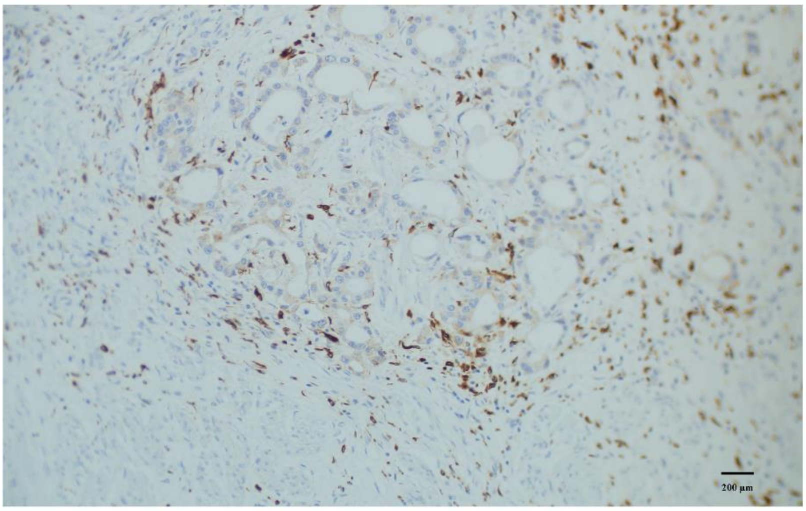

High CD68+ expression

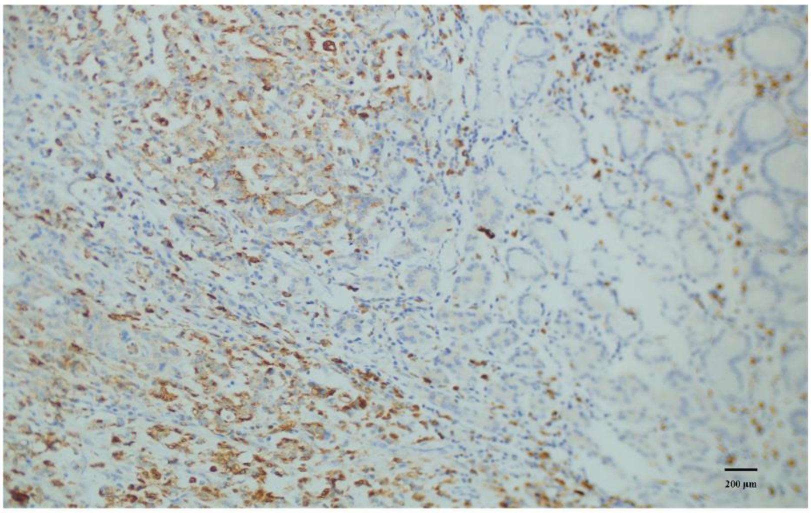

Fig.S1

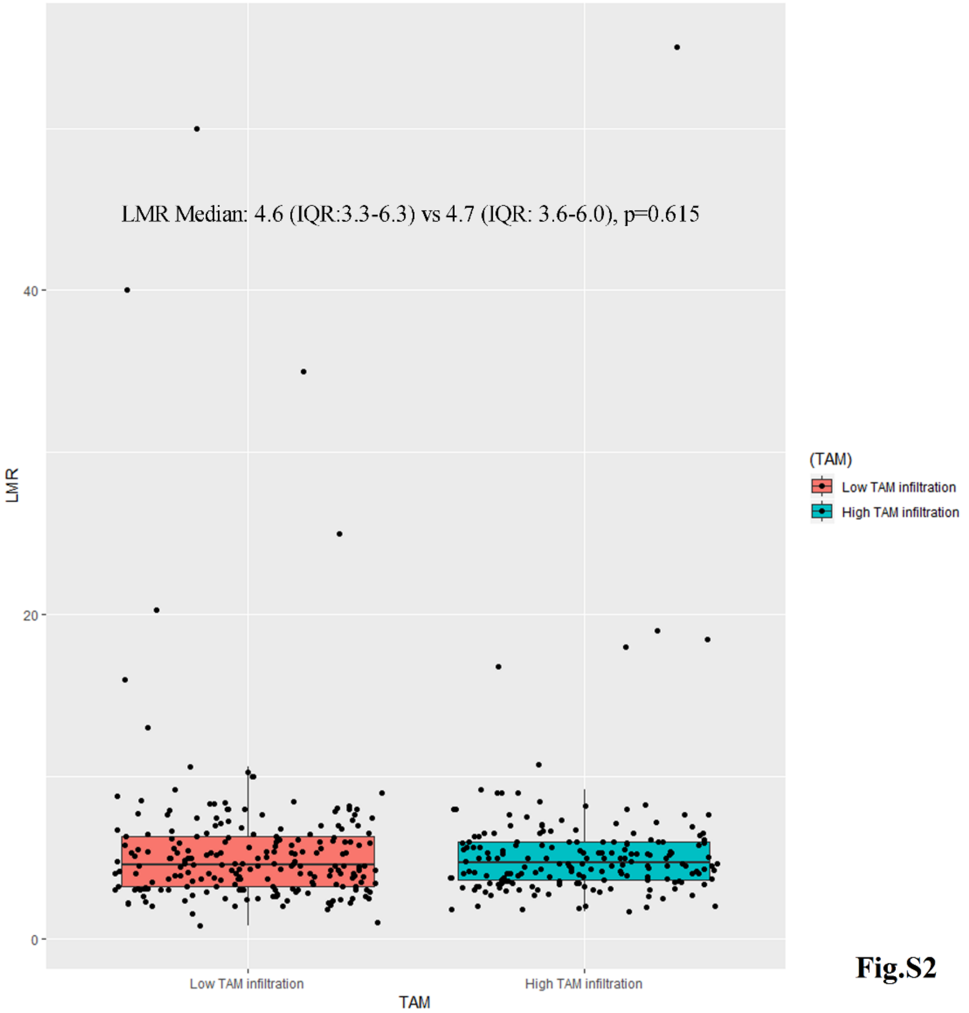
LMR Median: 4.6 (IQR:3.3-6.3) vs 4.7 (IQR: 3.6-6.0), p=0.615
LMR
40
20
0
Low TAM infiltration
High TAM infiltration
TAM
(TAM)
Low TAM infiltration
High TAM infiltration

Fig.S2

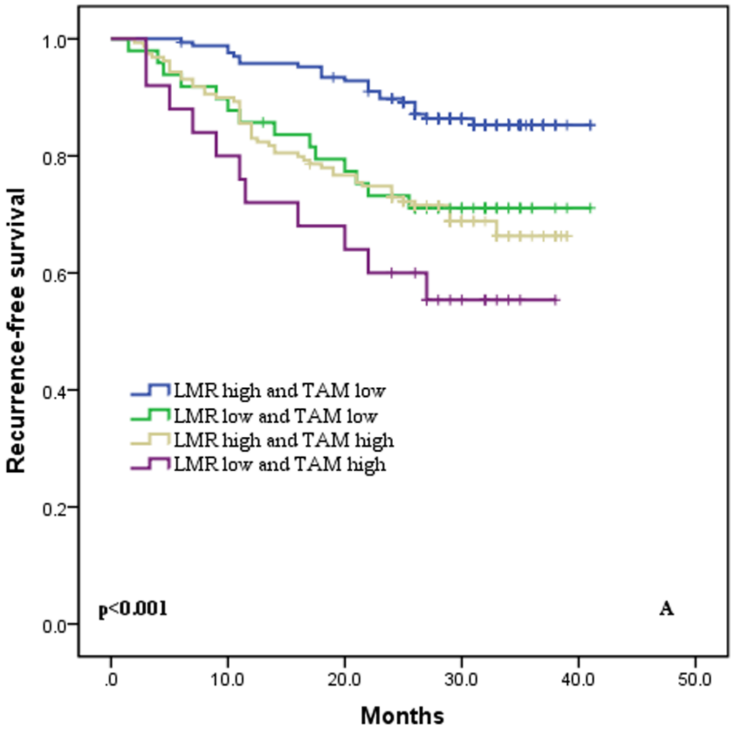
Recurrence-free survival
1.0
0.8
0.6
0.4
0.2
0.0
LMR high and TAM low
LMR low and TAM low
LMR high and TAM high
LMR low and TAM high
p<0.001
A
.0
10.0
20.0
30.0
40.0
50.0
Months

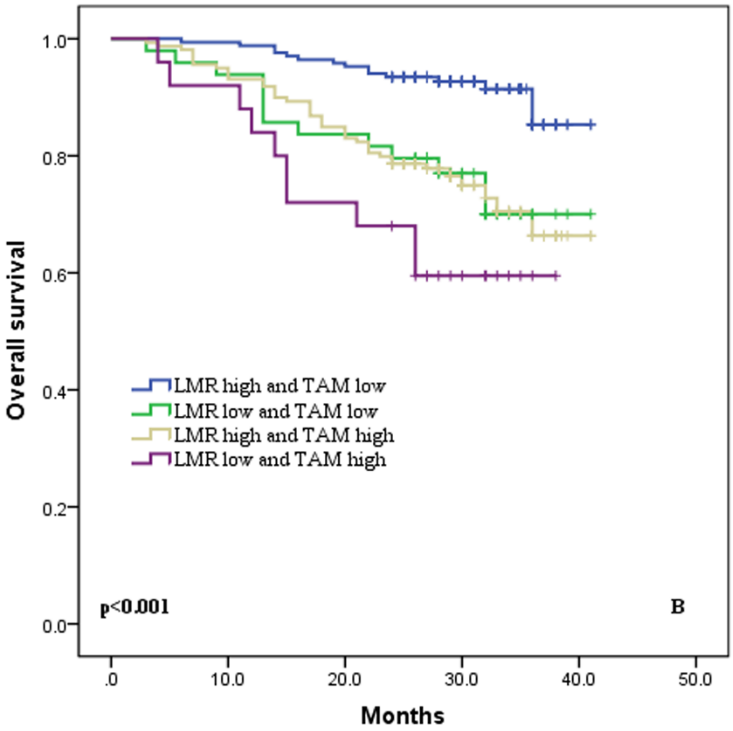
Overall survival
1.0
0.8
0.6
0.4
0.2
0.0
LMR high and TAM low
LMR low and TAM low
LMR high and TAM high
LMR low and TAM high
p<0.001
B
.0
10.0
20.0
30.0
40.0
50.0
Months

Fig. S3
